# Effect of Radial Shock Wave Therapy on Spasticity of the Upper Limb in Patients With Chronic Stroke: A Prospective, Randomized, Single Blind, Controlled Trial

**DOI:** 10.1097/MD.0000000000003544

**Published:** 2016-05-06

**Authors:** Tsung-Ying Li, Chih-Ya Chang, Yu-Ching Chou, Liang-Cheng Chen, Heng-Yi Chu, Shang-Lin Chiang, Shin-Tsu Chang, Yung-Tsan Wu

**Affiliations:** From the Department of Physical Medicine and Rehabilitation (T-YL, C-YC, L-CC, H-YC, S-LC, S-TC, Y-TW), Tri-Service General Hospital, School of Medicine; School of Public Health, National Defense Medical Center (Y-CC), Neihu District, Taipei; Department of Physical Medicine and Rehabilitation (L-CC), Hualien Armed Forces General Hospital, Hualien County; and Department of Rehabilitation (S-TC), Taichung Veterans General Hospital, Taiwan Boulevard, Taichung, Taiwan, Republic of China.

## Abstract

Recently, studies have reported that extracorporeal shock wave therapy (ESWT) is a safe, noninvasive, alternative treatment for spasticity. However, the effect of ESWT on spasticity cannot be determined, because most studies to date have enrolled small patient numbers and have lacked placebo-controlled groups and/or long-term follow-up. In addition, whether varying the number of ESWT sessions would affect the duration of the therapeutic effect has not been investigated in a single study. Hence, we performed a prospective, randomized, single blind, placebo-controlled study to investigate the long-term effect of radial ESWT (rESWT) in patients with poststroke spasticity and surveyed the outcome of functional activity.

Sixty patients were randomized into 3 groups. Group A patients received 1 session of rESWT per week for 3 consecutive weeks; group B patients received a single session of rESWT; group C patients received one session of sham rESWT per week for 3 consecutive weeks. The primary outcome was Modified Ashworth Scale of hand and wrist, whereas the secondary outcomes were Fugl-Meyer Assessment of hand function and wrist control. Evaluations were performed before the first rESWT treatment and immediately 1, 4, 8, 12, and 16 weeks after the last session of rESWT.

Compared to the control group, the significant reduction in spasticity of hand and wrist lasted at least 16 and 8 weeks in group A and B, respectively. Three sessions of rESWT had a longer-lasting effect than one session. Furthermore, the reduction in spasticity after 3 sessions of rESWT may be beneficial for hand function and wrist control and the effect was maintained for 16 and 12 weeks, respectively.

rESWT may be valuable in decreasing spasticity of the hand and wrist with accompanying enhancement of wrist control and hand function in chronic stroke patients.

## INTRODUCTION

Extracorporeal shock wave therapy (ESWT) is defined as a sequence of acoustic pulses characterized by high peak pressure (100 MPa), fast pressure rise (<10 ns), short duration (10 μs), and an energy density ranging from 0.003 to 0.890 mJ/mm.^1^ Different studies and clinical experiments have demonstrated the efficacy of ESWT in the treatment of musculoskeletal disorders such as chronic tendinopathies, calcific tendinitis of the shoulder, lateral epicondylitis, and plantar fasciitis, etc.^1^ The side effects of ESWT including aching, tingling, redness, or bruising are relatively rare and transitory.^1^

Radial ESWT (rESWT), a type of pneumatically generated shock wave, has a low to medium energy compared with traditional focused ESWT (fESWT). These unforced shock waves disperse eccentrically from the applicator tip without focusing the energy to a targeted spot. The penetrative depth is therefore less than that of fESWT (up to 3 vs 12 cm).^2^ A recent systematic review and meta-analysis reported potential advantages of rESWT over fESWT in patients with plantar fasciitis because rESWT has a larger treatment area, specific focusing is less important, it does not require additional local anesthesia, and it is cheaper.^2^

Spasticity is a common complication in patients with stroke and is defined as a velocity-dependent enhancement in muscle tone in response to passive stretching because of supraspinal disinhibition of stretch reflexes. The prevalence of spasticity is reported as 39% in patients with 1st-ever stroke after 12 months.^3^ The constant contraction of spastic muscles can produce pain, declined mobility, contractures, and skeletal deformities, which may limit the potential effect of rehabilitation.^4^ Common management of spasticity consists of passive stretching, splints, drug, phenol injection, and botulinum toxin (BTX) injection. However, current treatments of spasticity in poststroke survivors are often unsatisfactory.^5^

In recent years, studies have reported that ESWT is a safe, noninvasive, alternative treatment for spasticity that does not cause muscle weakness or unpleasant effects in patients with stroke,^6–15^ cerebral palsy,^16–19^ and multiple sclerosis.^20^ Although a recent small meta-analysis (including only 5 studies) reported that ESWT had a significant effect on improving spasticity 4 weeks after treatment compared with baseline in patients with brain injury,^21^ the effect of ESWT on spasticity cannot be determined because most studies to date have enrolled small patient numbers, and have lacked placebo-controlled groups and/or long-term follow-up. Among these studies, only 3 have included a placebo-controlled group in patients with cerebral palsy,^17^ stroke,^7^ and multiple sclerosis.^20^ To the best of our knowledge, only 1 study, without a control group, has applied rESWT for spasticity of the upper extremity in stroke patients.^14^ Whether varying the number of ESWT sessions would affect the duration of the therapeutic effect has not been investigated in a single study. Moreover, the general improvement in functional disability after reduction of spasticity via ESWT application to the upper limb has rarely been investigated in previous studies.

Hence, we performed a prospective, randomized, single blind, placebo-controlled study to investigate the long-term effect of rESWT in patients with poststroke spasticity and surveyed the outcome of functional activity.

## METHODS

### Study Design

This was a prospective, randomized, placebo-controlled, single-blind study conducted in a single medical center from April 2014 to May 2015. This study was reviewed and approved by the institutional review board of the Tri-Service General Hospital (No. 2-102-05-018) and all enrolled subjected gave their written, fully informed consent for the study. It was registered at ClinicalTrials.gov, trial NCT02221011.

### Randomization

The 60 enrolled patients were block randomized in a 1:1:1 ratio into 3 groups by an independent researcher using computer-generated randomization of study numbers (Microsoft Excel). Group A patients received 1 session of rESWT per week for 3 consecutive weeks; group B patients received a single session of rESWT; and group C patients received 1 session of sham rESWT per week for 3 consecutive weeks. During the study period, the dosage of antispastic medication was not adjusted and rehabilitation of the target area remained unchanged from 2 months prior to participation to the end of the follow-up period.

### Inclusion and Exclusion Criteria

We recruited patients with stable spasticity (no variability within 2 months before recruitment) in the wrist and hand (at least grade 1+ measured by the Modified Ashworth Scale [MAS]) at least 9 months after the onset of stroke to reduce the confounding effect of natural recovery.^22^ Patients with fixed contractures in the wrist or fingers, bilateral hemiplegia, or prior or planned treatment with phenol or alcohol nerve blocks, intrathecal baclofen, or BTX within the 6 months preceding the study were all excluded. Patients with malignant tumors, coagulopathy, pacemakers, or infections were also excluded.

### Shock Wave Therapy Instrumentation

Physio Shock Wave Therapy (Pagani Elettronica, Milano, Italy) was used for rESWT.^23^ The rESWT was focused in the flexor spastic muscles of the forearm, intrinsic muscles, and flexor digitorum tendon of the hand: 1500 shots with a pressure of 3.5 bar and frequency of 5 Hz were used to treat the flexor carpi ulnaris and radialis, mainly in the middle of the belly. In addition, 4000 shots with a pressure of 3 bar and frequency of 5 Hz were used diffusely for the intrinsic muscles and flexor digitorum tendon of the hand using an ultrasound pointer guide (Terason, t3000, Teratech, MA).^6,17^ The whole procedure is painless and does not require additional anesthesia or analgesic drugs. In the control group, the sham rESWT made the same sound but did not emit energy.

### Outcome Measurements

**FIGURE 1:**
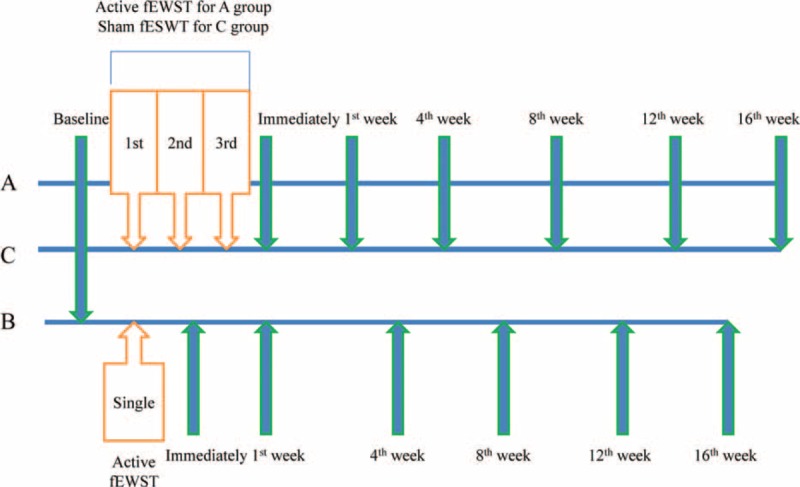
Timeline of treatment session with data collection in 3 groups. Group A patients received 1 session of radial extracorporeal shock wave therapy (rESWT) per week for 3 consecutive weeks; group B patients received a single session of rESWT; group C patients received 1 session of sham rESWT per week for 3 consecutive weeks. Evaluations were performed before the 1st rESWT treatment and immediately 1, 4, 8, 12, and 16 weeks after the last session of rESWT in each group (except hand function and wrist control immediately after rESWT).

Patients were examined by the same physiatrist, who was blinded to the randomization and treatment procedure. Evaluations were performed before the 1st rESWT treatment and immediately 1, 4, 8, 12, and 16 weeks after the last session of rESWT (Figure 1).

### Primary Outcome

#### Modified Ashworth Scale (MAS)

The MAS is extensively and reliably used in clinical practice and research to evaluate spasticity and is reported to have good validity in patients with chronic stroke.^24^ The scale is graded in 6 stages (0, no increase in tone; 1, slightly increased tone, giving a catch/release or minimal resistance at the end range of motion [ROM]; 1^+^, slightly increased tone, giving a catch followed by minimal resistance throughout the remainder [less than half] of the ROM; 2, more markedly increased tone through most of the ROM but affected part easily moved; 3, considerably increased tone and passive movement difficult; and 4, limb rigid in flexion or extension). For convenience of statistical analysis, MAS grade 1^+^ was point 2; grades 2, 3, and 4 were respectively matched to 3, 4, and 5.^11,14^

### Secondary Outcomes

#### Fugl-Meyer Assessment (FMA)

The FMA assesses motor function recovery after stroke and consists of 33 and 17 performance items in the upper and lower limbs, respectively. The scores range from 0 (unable to perform), to 1 (partial ability to perform), to 2 (near normal ability to perform).^25^ The items that measure wrist control and hand function have been revealed to have excellent intrarater reliability and high interrater reliability.^26^

#### Sample Size

To reduce a type II error and increase the power, a preliminary power analysis using G∗power 3.1.9.2 computer program, based on a one-way analysis of variance (ANOVA) test with comparison of 3 groups; power (1 − β) = 0.85; α = 0.05; effect size = 0.45, indicated that a total sample of 60 people would be needed.^27^

### Data Analysis

Statistical analyses were performed using the IBM SPSS statistics version 22 (IBM SPSS statistics 22). Demographic data were analyzed by the one-way ANOVA test for continuous data and the Chi-square test for categorical data. The differences between 3 groups were investigated using the one-way ANOVA followed by the Bonferroni post hoc tests. Statistical significance was set at *P* < 0.05.

## RESULTS

**FIGURE 2:**
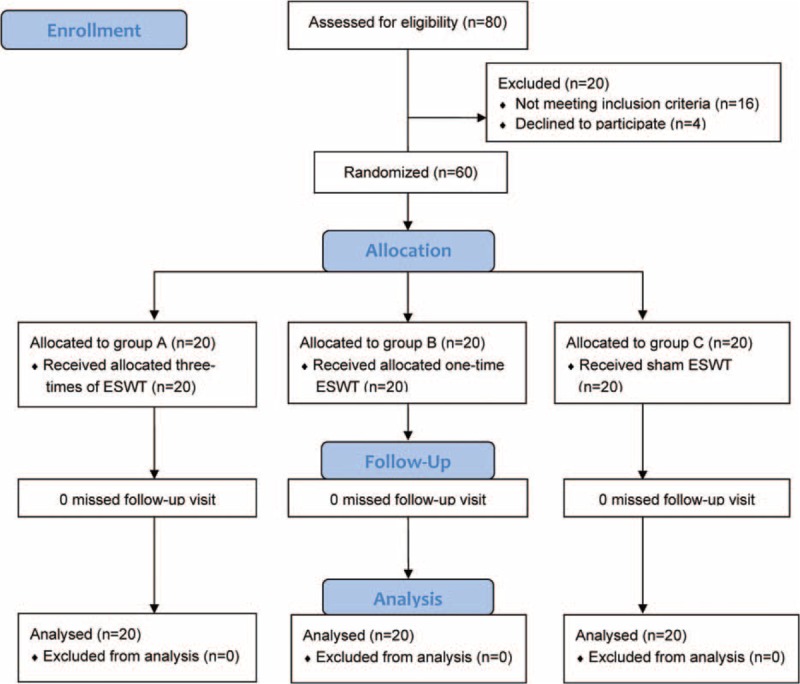
CONSORT flow diagram.

A total of 60 patients completed the study and each group consisted of 20 cases (Figure 2 for the flow diagram of enrollment). Total 6, 8, and 7 patients took antispasticity medications in group A, B, and C, respectively. There were no significant differences in baseline demographic characteristics between the groups (Table 1). No serious side-effects or complications after rESWT were observed in any of the 3 groups during the study period.

**TABLE 1:**
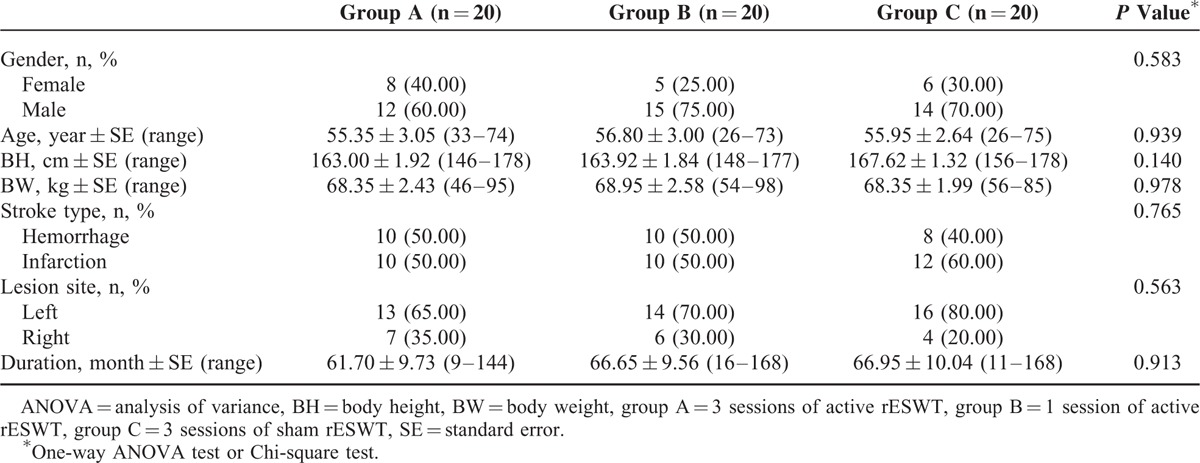
Baseline Demographic and Clinical Characteristics of Study Participants

**FIGURE 3:**
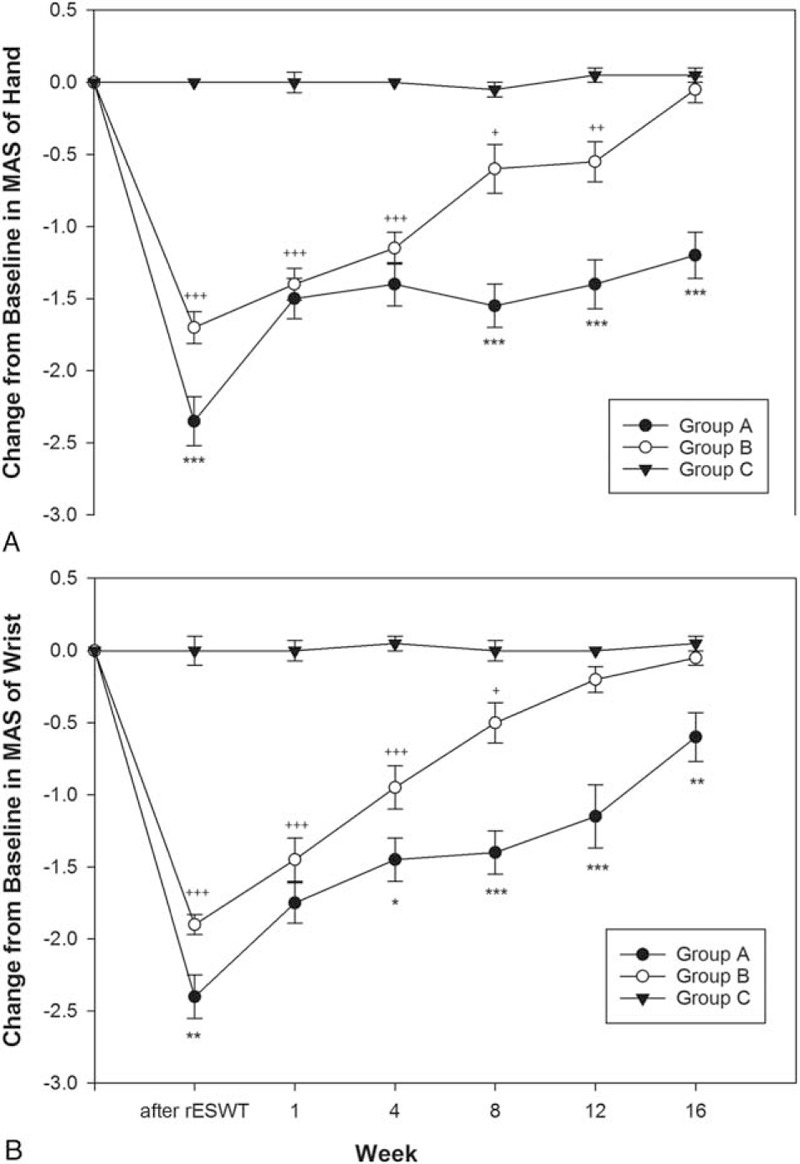
Mean of change from baseline in MAS in all groups (mean ± standard error). (A) MAS of hand: group B had significant improvement compared with group C until week 12. The differences between group A and group B reach significant at most of the observed time-points (except week 1 and week 4). (B) MAS of wrist: group B had significant improvement compared with group C until week 8. The differences between group A and group B reach significant at most of the observed time-points (except week 1). (^∗^*P* < 0.05, ^∗∗^*P* < 0.01, and ^∗∗∗^*P* < 0.001 mean group A vs B; ^+^*P* < 0.05, ^++^*P* < 0.01, and ^+++^*P* < 0.001 mean group B vs C. One-way ANOVA followed by the Bonferroni post hoc tests was used). ANOVA = analysis of variance, MAS = Modified Ashworth Scale.

Table 2 presents the difference of MAS scores of the wrist and hand before and after treatment. The difference of MAS-hand scores in group A were significantly larger than those of group C at all observed time-points and similar findings were noted in group B until week 12 (Table 2 and Figure 3A). Moreover, the differences in MAS-hand scores between group A and group B reach significant at most of the observed time-points (except week 1 and week 4) (Table 2 and Figure 3A). The difference of MAS-wrist scores in group A were significantly larger than those of group C at all observed time-points, and similar findings were noted in group B until week 8 (Table 2 and Figure 3B). Moreover, the differences in MAS-wrist scores between group A and group B reach significant at most of the observed time-points (except week 1) (Table 2 and Figure 3B).

**TABLE 2:**
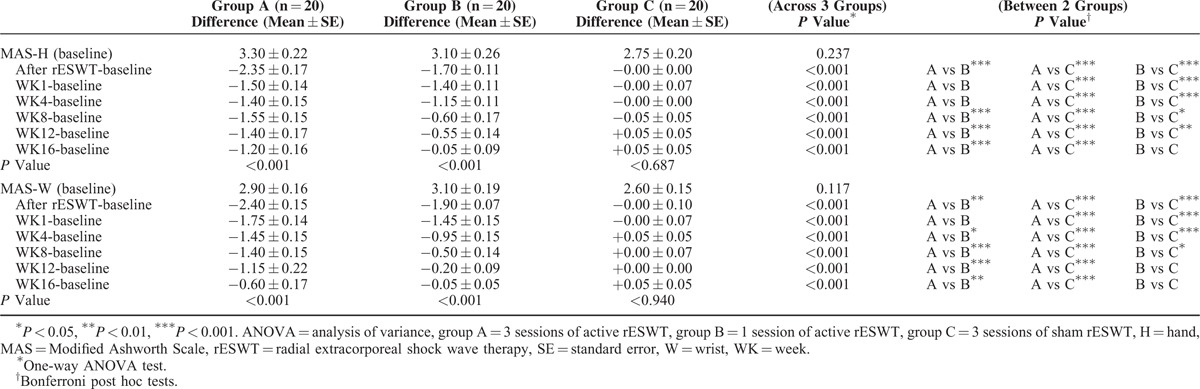
Mean and SE of Change From Baseline in Modified Ashworth Scale (MAS)

**FIGURE 4:**
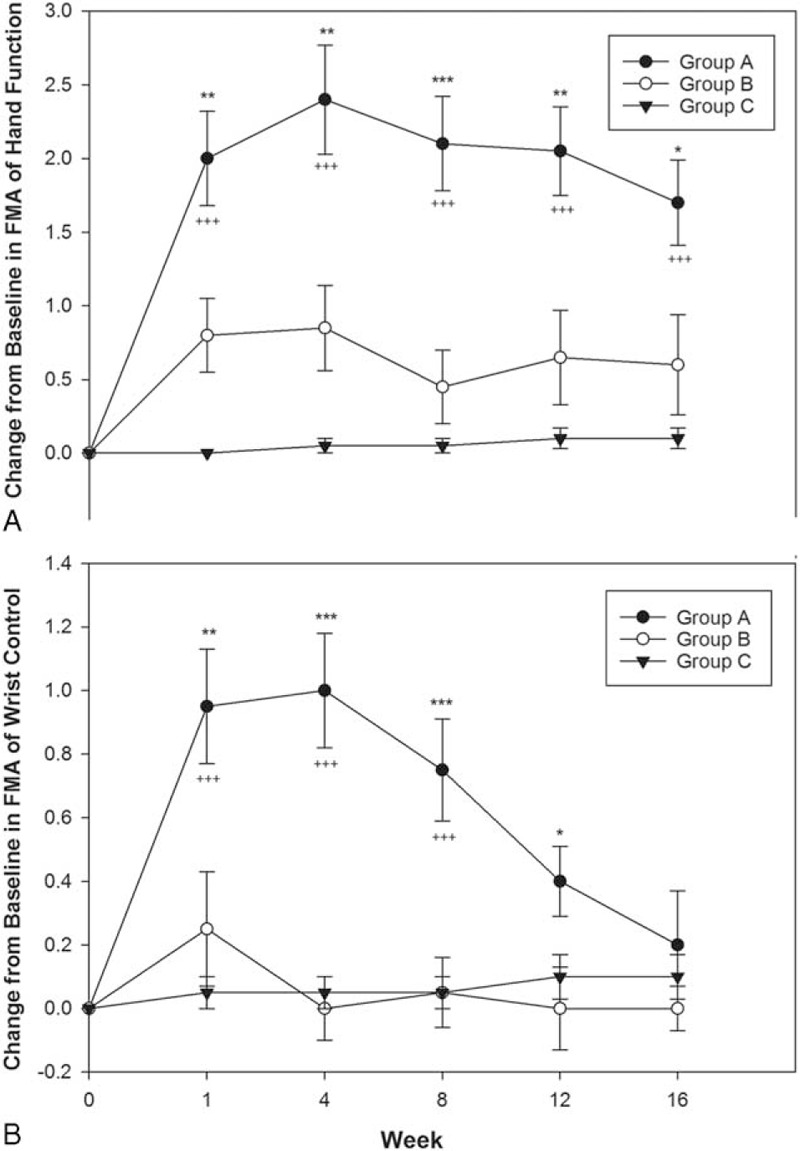
Mean of change from baseline in FMA in all groups (mean ± standard error). (A) FMA of hand function: group A had significant improvement compared with group B and C at all observed time-points. (B) FMA of wrist control: the difference in group A was significantly larger than those of group C until week 8 and those of group B until week 12. (^∗^*P* < 0.05, ^∗∗^*P* < 0.01, and ^∗∗∗^*P* < 0.001 mean group A vs B; ^+++^*P* < 0.001 mean group A vs C. One-way ANOVA followed by the Bonferroni post hoc tests was used). ANOVA = analysis of variance, FAS = Fugl-Meyer Assessment.

Table 3 presents the difference of FMA scores of hand function and wrist control before and after treatment. The difference of hand function scores in group A were significantly larger than those of group B and group C at all observed time-points (Table 3 and Figure 4A). Moreover, the difference of wrist control scores in group A were significantly larger than those of group C until week 8 and those of group B until week 12 (Table 3 and Figure 4B). However, no significant improvement of hand function and wrist control scores were observed in group B compared with group C (Table 3 and Figure 4).

**TABLE 3:**
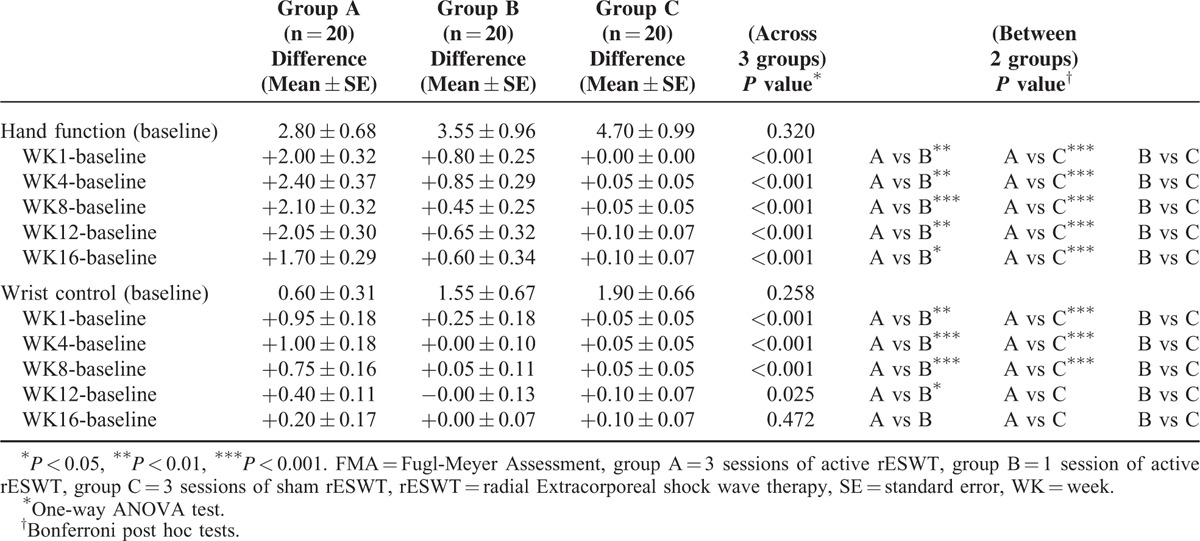
Mean and SE of Change From Baseline in Fugl-Meyer Assessment (FMA)

## DISCUSSION

To the best of our knowledge, the present study is the first prospective, randomized, single blind, placebo-controlled study to investigate the long-term effect of rESWT for the treatment of spasticity in patients with chronic stroke. Compared to the control group, groups receiving rESWT obtained a significant reduction in spasticity. After 3 sessions of rESWT, this reduction lasted at least 16 weeks; after 1 session the reduction lasted for 8 to 12 weeks. Three sessions of rESWT had a more noticeable and longer-lasting effect than 1 session, especially with regard to wrist spasticity. Furthermore, the reduction in spasticity after 3 sessions of rESWT may be beneficial for hand function and wrist control and the effect was maintained for 16 and 12 weeks, respectively.

Previous studies have demonstrated that ESWT has a positive effect on spasticity in patients with stroke, cerebral palsy, and multiple sclerosis, regardless of whether rESWT^14,16–18,20^ or fESWT^6,7,9–13,15,19^ is used. Outcomes and duration of effect varied across studies. Follow-up times were rarely longer than 3 months in published studies, except for 1 study which had a follow-up time of 6 months.^12^ Differences in the mechanism used to generate the shock wave, therapeutic energy, number of applications, target area, duration of spasticity, and patients’ age may have contributed to the varied duration of effect reported in previous studies (1 week to 6 months). Although 3 studies included a placebo-controlled group, short follow-up periods precluded investigation of the long-term effect of ESWT.^7,17,20^ The present result that rESWT decreases spasticity in the flexor muscles of the wrist and hand in patients with chronic stroke confirm the results of previous studies. This effect persists at least 16 weeks and 8 to 12 weeks after 3 sessions and 1 session of rESWT, respectively. Standard guidelines for the use of ESWT on soft tissue have not been established. Nevertheless, numerous studies used 2 or more sessions of ESWT for chronic tendinopathy; thus, clinical experience indicates repeated sessions of ESWT could be superior to a single application. Here, we report the 1st study investigating the effect of rESWT session number and confirm that repeated sessions of rESWT result in a more noticeable and longer-lasting effect.

Whether rESWT therapy is superior to fESWT in reducing spasticity is still uncertain. A previous study has reported that rESWT was superior to fESWT for treating plantar fasciitis due to its lower cost and enhanced effectiveness.^2^ Furthermore, rESWT is characterized by having a larger therapeutic area compared with fESWT, and specific focusing is less important. Hence, rESWT seems more suitable for treating spasticity because it can be applied to the whole muscle belly rather than a small spot in the muscle.^6,16^ To confirm this hypothesis, further study is needed in the future.

The mechanisms underlying the beneficial effects of ESWT on spasticity remain to be defined. Previous studies have proposed that ESWT may affect the production of nitric oxides (NO),^28^ decrease muscle fibrosis,^6^ modify spinal cord excitability,^6,29^ or affect the Golgi tendon organ^7^ or mechanical vibration.^6^ NO, which is generated by ESWT, is involved in neurotransmission, memory formation, and synaptic plasticity in the central nervous system, and in the formation of neuromuscular junctions in the peripheral nervous system.^30^ Hence, NO seems to play important roles in spasticity-reduction mechanisms. Kenmoku et al^31^ reported that the amplitude of the compound muscle action potential was significantly decreased immediately after ESWT and persisted for 8 weeks without delayed latency in an animal study. They also observed rapid degeneration of acetylcholine receptors after ESWT application and pointed out that these consequences were very similar to those of a neuromuscular transmission inhibitor like BTX. However, unlike BTX, no obvious weakness in the target muscle^31^ and no significant changes to F wave or H wave latency or amplitude were demonstrated in human studies after ESWT application.^6,9,20^ The effect on spinal excitability and Golgi tendon organs to suppress motor nerve excitability can be excluded as the main mechanism, although 1 recent study revealed a reduction of the H_max_/M_max_ ratio after ESWT (indicating a change in alpha motor neuron excitability).^15^

Although spinal excitability without long-lasting clinical or neurophysiologic effects could be caused by intermittent or constant mechanical vibration, this is unlikely to be the main contributor because it is temporary (lasting approximately several hours).^6^ The effects of ESWT on spinal excitability may support the idea that ESWT acts on nonreflex hypertonia. Recent studies have reported that abnormal stretch reflexes may not completely explain the development of spasticity.^32,33^ Chronic spasticity itself would further worsen joint resistance through fibrosis of inactive connective tissue due to structural and mechanical changes in the muscle.^34^ Moreover, reducing the stiffening of connective tissue caused by fibrosis of chronic hypertonic muscles would diminish spasticity.^6^ These associated mechanisms would explain the different durations of effect seen in group A and group B, because repeated treatments will be necessary to alter intrinsic stiffness. Further studies are needed to investigate this issue.

Studies investigating the effect of ESWT on spasticity seldom measure motor functional outcomes. Troncati et al^12^ was the 1st to use functional measurement in this field and showed a significant improvement in FMA of the upper limb in patients with stroke 6 months after ESWT treatment. However, the number of patients enrolled in the study was small (n = 10) and there was no control group. Moon et al^10^ revealed improved FMA scores in the lower limb after ESWT but the difference did not reach significance. In our findings, a significant improvement in FMA scores for hand function and wrist control after 3 sessions of rESWT was maintained for 16 and 12 weeks, respectively, compared with those of sham or 1 session of rESWT group. There was no significant improvement in FMA scores after 1 session of rESWT compared with sham control, indicating that repeated sessions of ESWT are necessary to ameliorate functional motricity. In contrast, other investigators did not observe any improvement in Barthel index, ambulation function, or Brunnstrom stage.^7,15,20^ This is hardly unexpected because of the poor sensitivity of global functional assessment scales in this context: they are more suited to assessing new motor learning that is practiced over a period. On the other hand, Simpson et al^35^ pointed out that the sensitivity of the FMA may be insufficient to detect alterations after treatment with BTX. Actually, the antispastic effect, especially the change in functional activity, of BTX might not be suitable in chronic hypertonic muscles because persistent spasticity can lead to further fibrosis of connective tissue.^34^ For this reason, ESWT (especially rESWT) may be more suitable in the treatment of chronic hypertonia and advanced effects on motricity compared with BTX. However, further studies are required.

Although many treatments of spasticity exist, it is still unsatisfactory in treating spasticity because each therapy has considerable risk of side effects. For example, systemically administered antispastic drugs may induce weakness of normal muscles and diminish with prolonged use.^36^ Chemical neurolysis with phenol wound cause dysaesthesia.^36^ In addition, repetitive injections of BTX might stimulate the formation of antibodies and the dosage is not always enough to treat rigorous and extensive spasticity.^37^ Compared with these conventional treatments, ESWT is a safe, effective, practical, easy-learning, and noninvasive method for relieving spasticity. Furthermore, repetitive or cyclic application of ESWT can also be considered because of rare side effects. However, further study is needed to determine which treatment has a greater effect.

The results of this study must be viewed in light of its limitations. First, the mechanism of rESWT for reducing spasticity was not evaluated in this study. Second, the number of cases was relatively small, although we had more subjects than most other published studies in this field. Third, the possible confounding effect of rehabilitation and antispastic medicine could not been ruled out. However, all patients were enrolled at least 9 months after stroke onset and had not had any changes to their existing physical programs or medicine for 2 months before participating. Moreover, the severity of spasticity was stable in all patients before they enrolled in the study. The scoring of the MAS is relatively objective and the evaluator was blinded to randomization. The significant improvement of spasticity after rESWT compared with the control group implies that decreases of spasticity and improvement of functional activity can be attributed to rESWT, not the rehabilitation programs or antispastic medicine. Fourth, the relatively low baseline of hand function and wrist control (low FMA score) in group A wound led to obviously potential improvement compared with other groups. Nevertheless, we found noticeably significant change (*P* < 0.001) compared A with B or A with C group in most follow-up time, the statistical difference of functional motricity after 3 sessions of rESWT might not be accidental. Finally, several questions, including the most effective intensity and number of ESWT sessions, remain unanswered and further studies need to be conducted in a larger number of patients and using multiple strategies.

In conclusion, our findings suggest that rESWT may be valuable in decreasing flexor spasticity of the hand and wrist with accompanying enhancement of hand function and wrist control in patients with chronic stroke. In addition, repetitive sessions of rESWT result in a longer-lasting and more noticeable effect, and are necessary for improving functional motricity.
